# Phyllosphere microbial associations improve plant reproductive success

**DOI:** 10.3389/fpls.2023.1273330

**Published:** 2023-12-08

**Authors:** Elijah C. Mehlferber, Reena Debray, Asa E. Conover, Julia K. Sherman, Griffin Kaulbach, Robert Reed, Kent F. McCue, Jon E. Ferrel, Rajnish Khanna, Britt Koskella

**Affiliations:** ^1^ Koskella Lab, University of California, Department of Integrative Biology, Berkeley, CA, United States; ^2^ Department of Environmental Studies, Haverford College, PA, United States; ^3^ i-Cultiver, Inc., Manteca, CA, United States; ^4^ Crop Improvement and Genetics Research Unit, Agricultural Research Service, Western Regional Research Center, United States Department of Agriculture, Albany, CA, United States; ^5^ Azomite Mineral Products, Inc., Nephi, UT, United States; ^6^ Department of Plant Biology, Carnegie Institution for Science, Stanford, CA, United States; ^7^ Chan Zuckerberg Biohub, San Francisco, CA, United States

**Keywords:** biostimulants, greenhouse growth, phyllosphere microbiome, plant probiotic, *Solanum lycopersicum* (tomato), synthetic microbiome, tomato yield

## Abstract

The above-ground (phyllosphere) plant microbiome is increasingly recognized as an important component of plant health. We hypothesized that phyllosphere bacterial recruitment may be disrupted in a greenhouse setting, and that adding a bacterial amendment would therefore benefit the health and growth of host plants. Using a newly developed synthetic phyllosphere bacterial microbiome for tomato (*Solanum lycopersicum*), we tested this hypothesis across multiple trials by manipulating microbial inoculation of leaves and measuring subsequent plant growth and reproductive success, comparing results from plants grown in both greenhouse and field settings. We confirmed that greenhouse-grown plants have a relatively depauperate phyllosphere bacterial microbiome, which both makes them an ideal system for testing the impact of phyllosphere communities on plant health and important targets for microbial amendments as we move towards increased agricultural sustainability. We find that the addition of the synthetic microbial community early in greenhouse growth leads to an increase in fruit production in this setting, implicating the phyllosphere microbiome as a key component of plant fitness and emphasizing the role that these bacterial microbiomes likely play in the ecology and evolution of plant communities.

## 1 Introduction

Microbial associations have been shown to be critical in the development and functioning of plant and animal host organisms (Shin et al., 2011; Wagner et al., 2014). For plants, there exists a wealth of data on how root and soil-associated microbial communities can shape plant growth, competition with neighbors, disease resistance, and nutrient uptake (Berendsen et al., 2012; Finkel et al., 2020; Qi et al., 2022). For example, in Arabidopsis the composition of their soil-based communities influences the plant’s resistance to drought stress (Zolla et al., 2013), while community diversity has been linked to nutrient uptake, with positive consequences for plant growth (Weidner et al., 2015). In contrast to the well-defined role of below-ground plant-associated communities, less is known about the importance of bacteria inhabiting the above-ground portion of the plant, the phyllosphere.

The phyllosphere microbiome is inhabited by a diverse consortium of bacteria, with densities ranging from 10^6^ to 10^7^ cells per square centimeter (Lindow and Brandl, 2003), as well as fungi, archaea, viruses (Sivakumar et al., 2020). These epiphytic microbial communities are subject to a hostile environment, often encountering high levels of UV radiation, temperature fluctuations, and desiccation (Jacobs et al., 2005; Beattie, 2011). Thus far, the investigation of phyllosphere microbiome impacts on plant health has generally been limited to their role in protection from disease, such as in cases of pear fire blight (Mercier and Lindow, 2001), tobacco wildfire disease (Qin et al., 2019), as well as *Pseudomonas syringae* associated diseases (Innerebner et al., 2011; Morella et al., 2019; Shalev et al., 2022). Although some evidence suggests that microbial communities can have key functions beyond disease resistance (Stone et al., 2018), for example through nitrogen fixation (Fürnkranz et al., 2008; Zhang et al., 2022) or the production of growth-regulating signals (Madhaiyan et al., 2006), there remains limited direct evidence for their role in plant fitness more broadly, such as through growth, flower, fruit, or seed production (reproductive success) (Saleem et al., 2017).

Most phyllosphere-inhabiting bacteria are believed to arrive on above-ground plant tissues via aerial transmission, including wind and rain (Vorholt, 2012; Ottesen et al., 2016), and much of this transmission is likely to originate from neighboring plants (Lajoie and Kembel, 2021; Meyer et al., 2022), though it is unclear if endospheric bacteria arrive in this way. Greenhouse-grown plants are expected to be relatively isolated from microbial dispersal via these channels, and indeed have been shown to develop communities distinct from those developing outdoors (Maignien et al., 2014). Since greenhouse plants are typically grown in commercial potting mixes, it is also unlikely that they have the full breadth of bacteria available for recruitment from the soil reservoir (Knief et al., 2010). Given that greenhouse-grown plants are likely depauperate in their microbial associations, they could provide a unique opportunity to understand the importance of phyllosphere bacteria by re-introducing these communities in a controlled environment.

One promising avenue for investigating the causative effects of plant-microbiota interactions in plant health is using synthetic bacterial communities. Ideally, these synthetic communities represent the phylogenetic diversity of natural phyllosphere communities, but at a tractable level of complexity, allowing for repeatable experimentation. This approach has been used to investigate a variety of plant-microbial interactions (Bodenhausen et al., 2014; Bai et al., 2015; Hu et al., 2016; Castrillo et al., 2017; Berg and Koskella, 2018; Carlström et al., 2019; Schäfer et al., 2022), but these synthetic communities also hold great potential as microbial ‘probiotics’ or biostimulants (organisms that enhance plant growth through mechanisms other than nutrient or pesticide addition; du Jardin, 2015). The addition of beneficial bacteria would be especially useful in environments where microbial diversity is otherwise reduced and/or where host-microbiome associations have been disrupted by, for example, pathogen establishment or antimicrobial treatments.

We examine this question using a defined set of naturally occurring bacteria to establish a synthetic community (herein referred to as ‘PhylloStart’) that we developed based on observed diversity of bacterial species residing on field-grown tomato plants. To investigate interactions between microbial associations and nutrient status we included a commercially available micronutrient supplement (Azomite^®^) at various concentrations. We applied PhylloStart to aerial plant tissues during the first weeks of growth post-germination to mirror early life microbiome establishment and tested, across multiple trials, bacterial growth on leaves, disease resistance against a common bacterial tomato pathogen, and tomato fruit yield. We observed a significant increase in reproductive success (as measured by fruit production) in treated plants relative to controls. In line with the idea that phyllosphere recruitment is likely limited in the greenhouse, we find that field-transplanted plants sprayed pre-transplantation with PhylloStart did not show altered fruit yield in response to amendment. Overall, our results demonstrate that, unless supplemented, phyllosphere bacterial communities establish poorly under common greenhouse growth conditions and highlight the underappreciated role of the above-ground microbiome in shaping plant fitness.

## 2 Materials and methods

### 2.1 Plant generation

Seeds of tomato (*Solanum lycopersicum*) variety ‘Moneymaker’ were surface sterilized by shaking in a solution of sodium hypochlorite and 2% Tween 20 (1:2) for 20 min, followed by two rinses with filter-sterilized H_2_O. Seeds were sowed in trays with Sunshine Mix #1 (Sun Gro; Canadian Sphagnum peat moss, perlite, dolomite) and germinated in the greenhouse. When seedlings were ~4 inches tall, they were transplanted into pots and distributed across the greenhouse in a randomized design, where they were grown for the remainder of their development (20 weeks in the first trial, 19 weeks in the second trial and 24 weeks in the third trial) under controlled conditions with supplemented lights to maintain long days and fans to control temperature fluctuations. Plants were grown in the Greenhouse facility at the USDA Plant Gene Expression Center greenhouse in Albany, CA. Nutrient supplementation consisting of Peters Professional 20/20/20 water-soluble fertilizer was applied (1:64 ppm) once per week. A disease suppression program with Floramite and Decathlon at a rate of 1/4 tsp per gallon was applied through a controlled sprayer at the rate of 1 to 2 gal per 100 plants. Plants in the field trial were started in the greenhouse and received the same treatment as the plants in the third trial until 7 weeks, when they were moved outside to harden, and transplanted into the Oxford Tract field at UC Berkeley at 8 weeks. After transplanting into the field, plants were watered once a week on a drip system for approximately 6 hours, with 20-20-20 water-soluble fertilizer at a rate of 0.93 lb/ac. The drip irrigation prevented water splashing onto aerial tissues and thus reduced transmission of bacteria into soil/roots. Powdery mildew, a common nuisance in greenhouse environments was detected during the second trial and treated, as per standard protocols, by pruning infected tissue. The plants were randomly dispersed throughout the greenhouse, and regardless of location or treatment we did not see a noticeable difference in presence of powdery mildew, and plants were pruned equally across treatments.

### 2.2 Bacterial strains

PhylloStart was designed to mimic the composition of a field-grown tomato phyllosphere bacterial community. The community was designed based on community sequences from tomato plants in the Student Organic Farm at UC Davis (**Supplementary Table 1**). Isolates were collected to be representative at the family level of bacterial species found above 0.1%, and were collected directly from the leaves of tomato plants grown at the Student Organic Farm or, in 3 cases (*Pseudomonas asturiensis*, *Pseudomonas rhodesiae*, and *Pantoea allii*), from the endpoint of a greenhouse selection experiment (Morella et al., 2020). Leaf wash was initially plated on King’s Broth (KB) and LB (Lysogeny Broth) agar plates, followed by MacConkey, and 1% Tryptic Soy agar plates. Individual colonies were selected, amplified, and sequenced at the 16S rRNA locus. Included isolates represent 97.8% of the total bacterial relative abundance in our field sequencing at the family level, with families Enterobacteriaceae, Oxalobacteraceae, Pseudomonadaceae, Bacillaceae, Microbacteriaceae, and a member of Brevibacteriaceae that was identified at high prevalence from later field samples. In total, 16 species were selected (out of the 93 screened isolates), with several members representing species level variation within the selected families. Information on the identity of the PhylloStart synthetic community is available in **Supplementary Table 2**.

### 2.3 Phyllostart application

**Figure 1:**
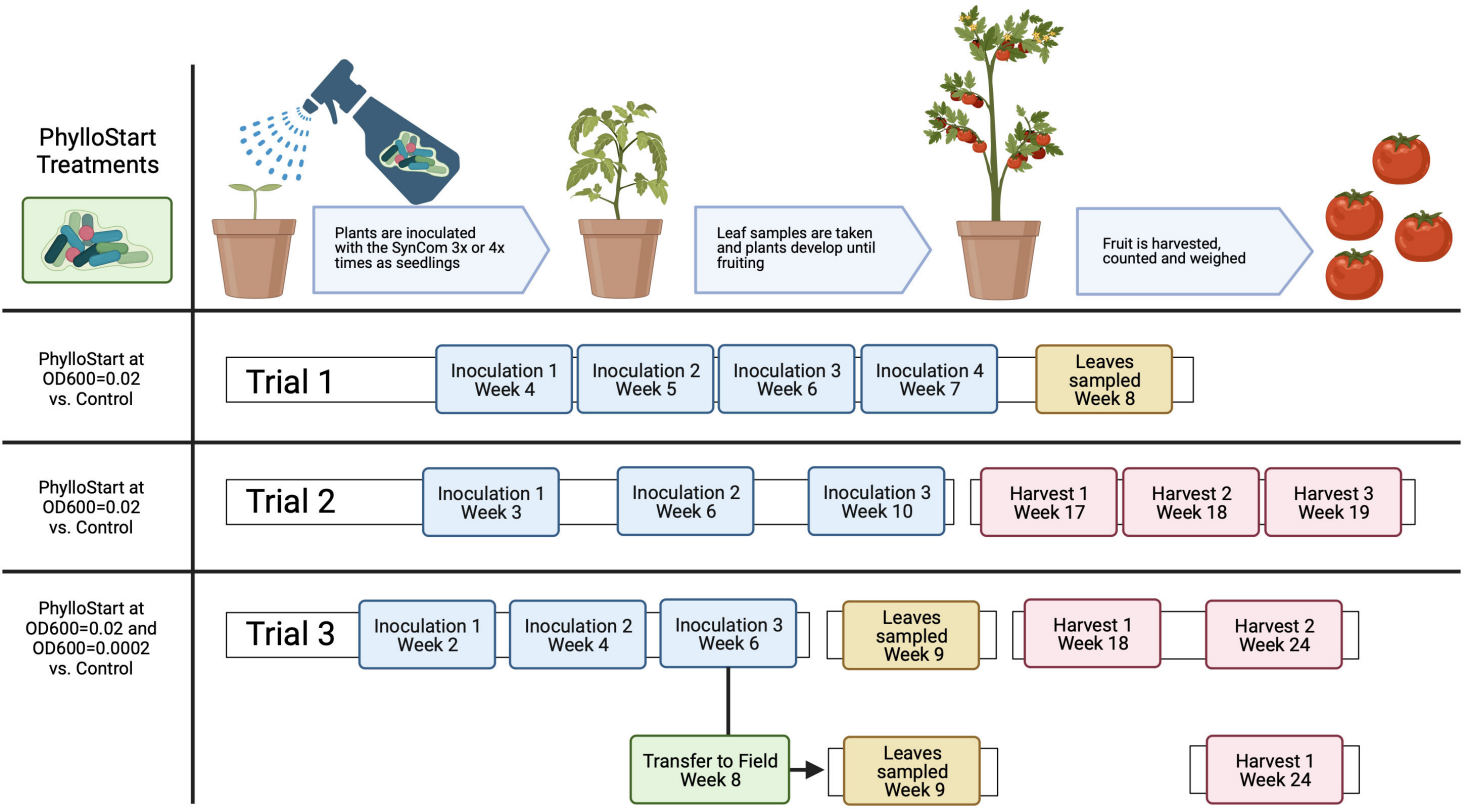
Experimental design for the greenhouse and field experiments with PhylloStart treatments and inoculation timing. See **Supplementary Figure 1** for more details on Azomite treatments.

For preparation of the PhylloStart consortia, strains were grown for three days at 28°C on a media shaker in KB broth. Cultures were then spun down for 10min at 2500g, and the supernatant was replaced with fresh KB. The optical density at 600nm (OD_600_) of each sample was read and the volume of each sample necessary to yield a concentration of 0.2 was calculated. Samples were mixed to yield this concentration and the suspension was frozen in 50/50 KB/glycerol at -80°C until inoculation. On the day of inoculation, the community was thawed, pelleted as above, and resuspended at a concentration of OD_600 = _0.02 in sterile 10 mM MgCl_2_ buffer with 0.01% Silwet surfactant. For the third greenhouse trial and the field trial we included two concentrations of PhylloStart, one at OD_600 = _0.02 and another diluted 100-fold. Plants were sprayed with either PhylloStart or sterile MgCl_2_ with Silwet onto both sides of all leaves until runoff (which was minimized to reduce any possible movement to soil). Inoculation timing varied among experiments; in the first trial plants were inoculated at weeks 4, 5 and 6, in the second trial at weeks 3, 6 and 10, and in the third trial at weeks 2, 4, and 6 (**Figure 1**).

### 2.4 Azomite application

Given the reduced nutrient composition of the potting mix used to grow plants, we supplemented soil with a commercial micronutrient product, Azomite^®^ (Azomite Mineral Products, Inc., UT) in order to determine whether PhylloStart had a nutrient-dependent impact. Azomite^®^ is a soil additive and fertilizer derived from volcanic ash that has been shown to increase the growth and yield of tomato plants (Noorani Azad et al., 2016; Mehlferber et al., 2022a). In the first greenhouse trial, we used three Azomite treatments: 5% wt/wt Azomite Granular during sowing and transplanting (n=10), 1g of Azomite Ultrafine applied after transplanting at the base of the plant at 7, 9, and 12 weeks after sowing (n=10), or both Azomite Granular and Azomite Ultrafine applied as described (n=10). This trial included a control treatment with no Azomite or PhylloStart (n=10), as well as a PhylloStart only treatment (n=10) and a treatment with both PhylloStart and Azomite Granular and Ultrafine (n=10). In the second greenhouse trial, we applied Azomite Granular to all Azomite treated plants while modifying the Azomite Ultrafine concentration using 1, 2 and 3 grams (n=3 with PhylloStart and n=3 without PhylloStart at each concentration), as well as a control treatment that did not receive either Azomite or PhylloStart (n=3). In the third greenhouse trial and the field trial, we included treatments with Azomite Granular and Ultrafine, using 1 and 2 grams in the greenhouse, and 1 and 3 grams in the field and either a low, or high dose of PhylloStart (n=4 for each), a PhylloStart only treatment at both concentrations (n=6 for each), and a control treatment (n=6). A second field trial was performed at UC Davis to confirm the results from our initial field trial, using the same methods as previously described, with the following treatments; PhylloStart (n=6), Control (n=6), PhylloStart with 3 grams of Azomite (n=6) and Control with 3 grams of Azomite (n=6). For a detailed overview of all treatments and replicates across experiments see **Supplementary Figure 1**.

### 2.5 Plant harvest

Tomatoes were harvested and their number and weight were recorded multiple times per plant from onset of fruit production to plant termination in the greenhouse, at weeks 17, 18 and 19 in the second trial, and weeks 18 and 24 in the third trial. These metrics were measured only once after harvest from each individual plant grown in the field, at week 24. Tomatoes were weighed individually in trial 2, and as total harvested weight per plant in trial 3, as described (**Supplementary Figure 2**).

### 2.6 Pathogen protection experiment

To determine whether PhylloStart conferred pathogen resistance to plants, tomato seeds were prepared as described above, then germinated onto 1% water-agar plates. After 1 week, seedlings were transferred to individual pots containing autoclaved calcined clay medium (Profile Porous Ceramic Greens Grade, Sierra Pacific Turf Supply). In this experiment we focus on a specific nutrient, Phosphorous, using 960 mg of organic fertilizer (0-11-0 Seabird Guano, Down to Earth) which was added to each pot at the transplant stage. Plants were randomized with respect to treatment and maintained in a growth chamber at a 15 h day:9 h night cycle for the duration of the experiment. PhylloStart was applied (as above) to leaves at a concentration of OD_600 = _0.02 with 0.01% Silwet surfactant on three-week-old plants. One week after spraying, an overnight culture of *Pseudomonas syringae* pathovar tomato PT23 was pelleted and diluted in 10 mM MgCl_2_ to a concentration of OD_600 = _0.0002 and inoculated into three leaves per plant via blunt-end syringe inoculation. At 24 hours post-infection, three hole punches (6-mm diameter) were taken from each inoculated leaf (9 total leaf discs per plant). Leaf discs were homogenized in 1 mL 10 mM MgCl_2_ in a FastPrep-24 5G sample disruption instrument at 4.0 m/s for 40 seconds. *Pseudomonas syringae* population density on leaves was obtained through colony forming unit (CFU) plating.

### 2.7 Phyllosphere bacterial microbiome sampling

Leaves were sampled in the first and third trials to assay the composition of the epiphytic phyllosphere community. In each case, 5 leaves were collected into 50ml conical tubes from random locations across each plant, in the first trial leaves were collected a week after the last inoculation, when the plants were 8 weeks old, while in the third trial they were collected three weeks after the last inoculation, when the plants were 9 weeks old to determine how much of the community persisted over time. These leaves were weighed and 40ml of sterile 10mM MgCl_2_ was added, then sonicated for 10 minutes, followed by five seconds of vortexing. The bacteria were pelleted, the supernatant was removed, and samples were frozen at -80°C until DNA extraction and sequencing.

### 2.8 DNA extractions, qPCR, 16S rRNA amplification, and sequencing

DNA extraction and sequencing was performed by the company Microbiome Insights using the following protocols. Bacterial pellets were placed into a MoBio PowerMag Soil DNA Isolation Bead Plate. DNA was extracted following MoBio’s instructions on a KingFisher robot. For qPCR, bacteria-specific forward primers (300nM 27F, 5′ -AGAGTTTGATCCTGGCTCAG-3′) coupled to reverse primers (300nM 519R, 5′ -ATTACCGCGGCTGCTGG-3′) were used to amplify bacterial 16S rRNA. 20μl reactions using iQ SYBR Green Supermix (BioRad), with 10µl Supermix, 0.6µl Primer F, 0.6µl Primer R, 6.8µl H_2_O and 2µl template, were run on Applied Biosystems StepOne Plus instrument in triplicate using the following cycle conditions; 95°C for 3min., 95°C for 20sec., 55°C for 20sec., 72°C for 30sec., return to step two 45 times. For standards, the full-length bacterial 16S rRNA gene was cloned into a pCR4-TOPO vector, with Kanamycin-Ampicillin resistance. The total plasmid fragment size is expected to be 5556 bp. A bacterial standard was prepared via 10-fold serial dilutions, and the copy number of 16S rDNA was determined as follows: Copy#=(DNA wt. x 6.02E23)/(Fragment Size x 660 x 1E9). Linear regression was used to determine copy numbers, based on the cycle threshold (Ct) of standards. Reaction specificity was assessed using a melt curve from 55°C to 95°C, held at 0.5°C increment for 1s.

For 16S rRNA amplification and sequencing, bacterial 16S rRNA genes were PCR-amplified with dual-barcoded primers targeting the V4 region (515F 5’-GTGCCAGCMGCCGCGGTAA-3’, and 806R 5’-GGACTACHVGGGTWTCTAAT-3’), as per the protocol of Kozich et al., 2013. Amplicons were sequenced with an Illumina MiSeq using the 300-bp paired-end kit (v.3). The potential for contamination was addressed by co-sequencing DNA amplified from specimens and from template-free controls (negative control) and extraction kit reagents processed the same way as the specimens. A positive control from samples consisting of cloned SUP05 DNA, was also included. The only modification to this standard protocol was the addition of peptide nucleic acid (PNA) PCR clamps according to the method developed in Lundberg et al., 2013. In brief, mPNA and pPNA were added into the PCR step during library prep at a concentration of 5µM per PNA to reduce amplification of mitochondrial and choroplast DNA, respectively. The PCR reaction was then modified with the addition of a PNA annealing step at 78°C for 10s.

### 2.9 Data analysis

Paired-end reads were filtered and trimmed to 230(F) and 160(R) base pairs (bps), using DADA2 with default parameters (Callahan et al., 2016). Following denoising, merging reads and removing chimeras, DADA2 was used to infer amplicon sequence variants (ASVs), which are analogous to operational taxonomic units (OTUs), and taxonomy was assigned using the DADA2-trained SILVA database. Using DNA extraction and PCR negative controls from 16S rRNA sequencing, the *decontam* package was implemented using default settings to identify and remove potential contamination from the samples (Davis et al., 2018). The assigned ASVs, read count data, and sample metadata were combined in a *phyloseq* object (McMurdie and Holmes, 2013) for downstream analyses. The *phyloseq* package was used to calculate beta diversity (using Bray-Curtis distance), and a permutational analysis (PERMANOVA) was performed on data rarified to 400 reads (Weiss et al., 2017) (to account for extraordinarily low read counts in untreated greenhouse samples) using the *adonis* function in the *vegan* package (Oksanen et al., 2022).

Fruit production was analyzed with two-way ANOVAs, and where appropriate, a Kruskal-Wallis test was used to control for unequal variances. Post-Hoc tests were performed with either Tukey’s HSD test or, when a Kruskal-Wallis was performed, Dunn’s Test. Tests were performed in R using the package *rstatix* (Kassambara, 2023). Tests for normality across residuals were performed for all linear mixed-effects model, and data was checked for outliers, normality, and homogeneity of variance prior to running the ANOVAs (**Supplementary Data 1**).

## 3 Results

### 3.1 PhylloStart bacteria colonize the plant phyllosphere (Trial 1)

**Figure 2:**
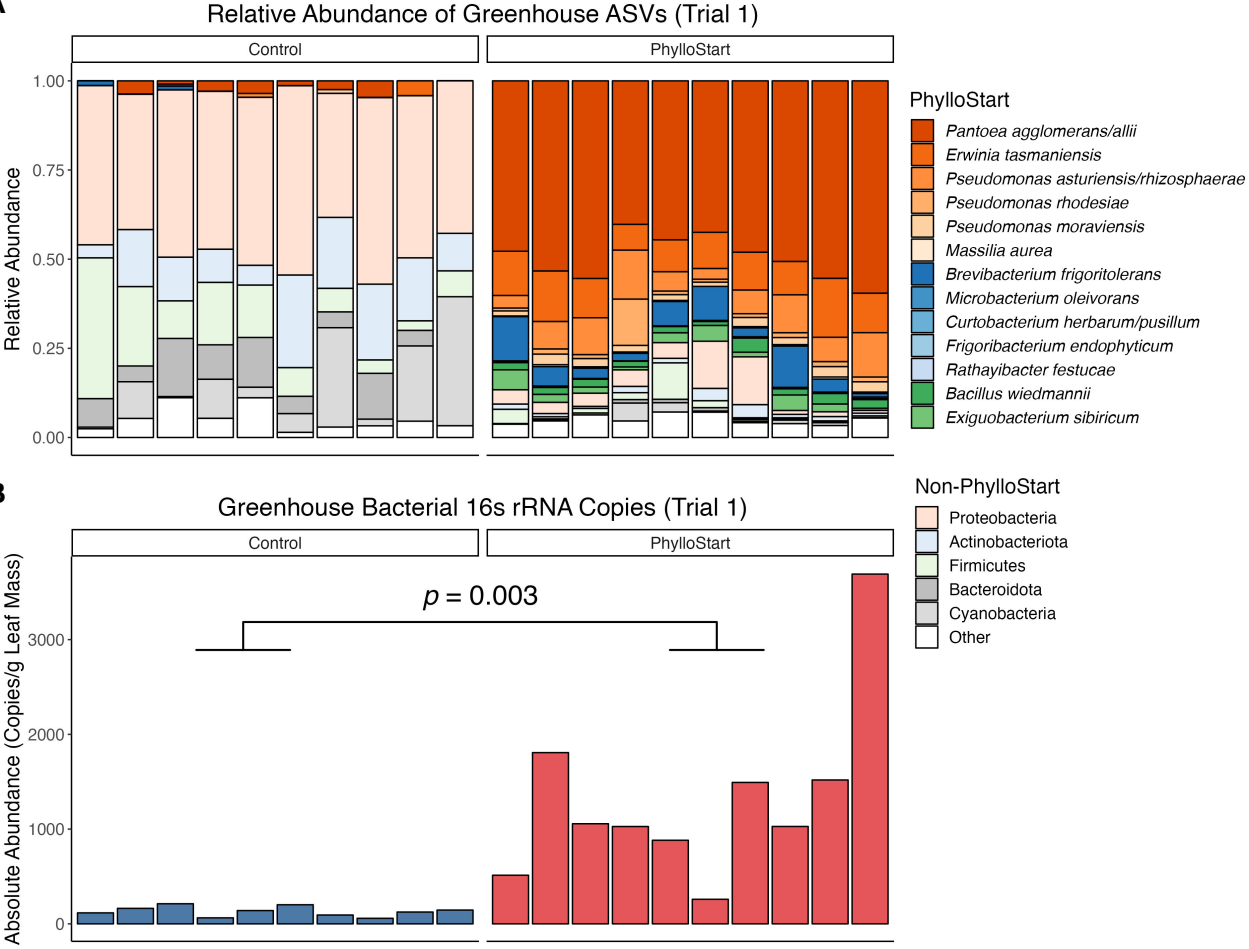
**(A)** Relative abundance of ASVs from greenhouse tomato leaves from the first trial. PhylloStart bacteria are highlighted individually (when their 16S rRNA sequences are distinct), while other bacteria are grouped by Phylum. Colors are consistent across PhylloStart and Phylum labels, with the red PhylloStart bacteria being Proteobacteria, the blue being Actinobacteriota, and the green being Firmicutes. **(B)** Number of bacterial 16S rRN copies detected on plants through qPCR sequencing, sampled one week after inoculation with the PhylloStart bacteria or a buffer control. Only the plants that have been inoculated with PhylloStart have an appreciable number of bacteria residing on their leaves.

We hypothesized that greenhouse-grown plants would be relatively depauperate in their microbial associations. To test this, we inoculated seedlings with the PhylloStart community and harvested leaves one week after the last application. There was no significant differences in community composition from plants treated with or without Azomite (Adonis PERMANOVA; *F*=0.92, *p=*0.543) and so for the sake of simplicity Azomite samples are not included in the Figure (see **Supplementary Figure 2** for full comparison). Supporting our hypothesis, we found a significantly higher abundance of bacteria on the leaves inoculated with PhylloStart than those treated with buffer (t-test; *t*=-3.97, *p*=0.003). Further, in the treated plants, the vast majority of the relative abundance of bacterial sequences were associated with PhylloStart members (**Figure 2**). Together, these data indicate that there was a robust initial representation of the PhylloStart community on the plant leaves, and that there is minimal development of leaf associated bacteria in the greenhouse in the absence of amendment.

### 3.2 PhylloStart inoculation and micronutrient supplementation independently increase tomato production (Trial 2)

**Figure 3:**
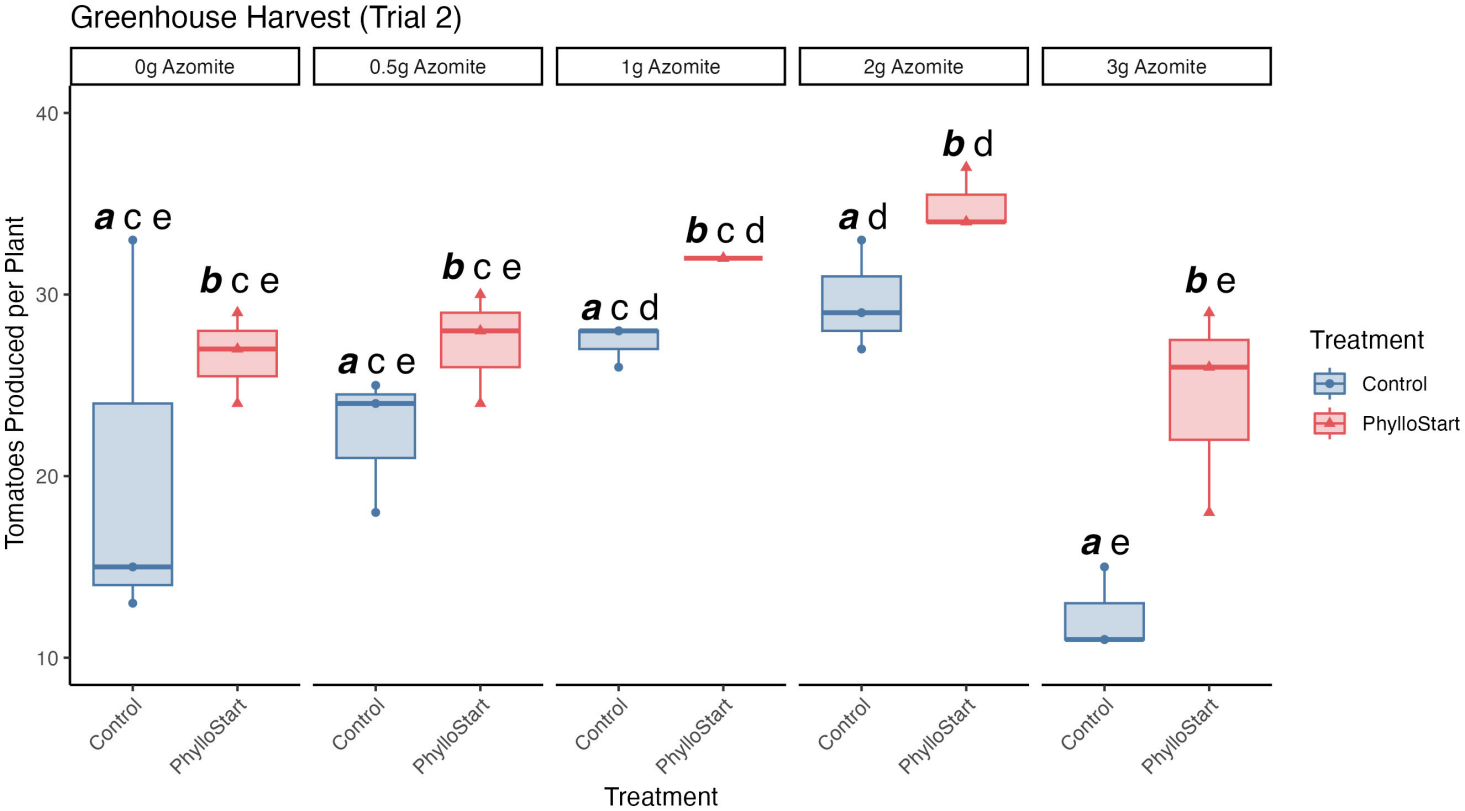
Total number of tomatoes produced across PhylloStart and micronutrient (Azomite) supplemented treatments from the second trial. Groups that are significantly different from each other are marked with different letters, while groups without significant differences will have the same letter. Emphasized lettering indicates comparisons across PhylloStart treatments (ie. *a*, *b*), while non-emphasized lettering indicates comparisons across Azomite treatments (ie. *c*, *d*, *e*). Both application of the PhylloStart bacteria (Control *a* vs. PhylloStart *b*) and Azomite addition (2g *d* vs 1g, 0.5g, and 0g *c*) lead to a significant increase in the total number of fruit produced. Of note, when adding Azomite at the highest level (3 grams), total productivity of the control plants was lower than when 2 grams was added (3g *e* vs. 2g *d*). This treatment did not significantly differ from the other Azomite treatments (0g, 0.5g, 1g, and 3g *e*) However, when these plants are additionally inoculated with PhylloStart they are rescued to at least the level of the control plants.

In order to determine if the PhylloStart bacteria may have an impact on fruit production, we repeated the experiment focusing on the total number of tomatoes produced across bacterial conditions and micronutrient supplement. In this experiment, we saw that PhylloStart treated plants produced, on average 29.1 (95% C.I. = 26.39, 31.74) tomatoes per plant, while control plants produced only 22.4 (95% C.I. = 18.08, 26.72) tomatoes per plant. Both Azomite application (two-way ANOVA; *F*(4,20)=8.851, *p=*<0.001, *ges* =0.639) and PhylloStart amendment (*F*(1,20)=16.447, *p=*<0.001, *ges* =0.451) were found to significantly impact fruit number, though their interaction did not (*F*(4,20)=0.687, *p=*0.61, *ges* =0.121). Tukey’s HSD Test for multiple comparisons uncovered significant differences between Control and PhylloStart treatments (*p=*<0.001, 95% C.I. = 3.36, 9.97), and several micronutrient treatments, with significant differences between 0-gram and 2-gram Azomite treatments (*p=*0.015, 95% C.I. = 1.38, 16.29) as well as between 0.5-gram and 2-gram Azomite treatments (*p=*0.048, 95% C.I. = 0.05, 14.95) (**Figure 3**). Interestingly, the highest level of micronutrient supplementation led to a significant decrease in yield across two comparisons; 1-gram compared to 3-gram Azomite treatments (*p=*0.001, 95% C.I.=-18.79, -3.88) and 2-gram compared to 3-gram Azomite treatments (*p=*<0.001, 95% C.I.=-21.45, -6.54) (**Figure 3**). At this concentration plants treated with PhylloStart were slightly (but not significantly) less affected than those treated with the micronutrient supplement alone (t-test, *t=*-3.38, *p=*0.052), indicating that the phyllosphere bacteria may have partially rescued the plants from this abiotic stress (**Figure 3**).

In order to rule out that the plants were producing more but smaller tomatoes, we measured both tomato number and weight throughout the course of the trials (**Supplementary Figure 3A**). We see no significant differences in the weights of tomatoes across PhylloStart treatments (Kruskal-Wallis test; *H*(1)=0.19, *p=*0.663). In contrast, there is a significant positive effect of Azomite treatment on tomato weight (*H*(1)=22.17, *p<*0.001). Dunn’s Test indicated that there was a significant increase in tomato weight in the 2-gram Azomite treatment (average weight 55.01 grams (95% C.I. = 51.52, 58.52)) compared to the control (average weight 45.97 grams (95% C.I. = 43.27, 48.67); Post-Hoc Dunn test, *Z=*3.016, *p=*0.02), a significant increase in tomato weight in the 2-gram Azomite treatment compared to the 0.5-gram Azomite treatment (average weight 47.02 grams (95% C.I. = 45.12, 48.93), *Z=*2.72, *p=*0.006), as well a significant decrease in tomato weight in the 3-gram Azomite treatment (average weight 41.05 grams (95% C.I. = 35.07, 47.05) compared to the 2-gram Azomite treatment; *Z=*-3.93, *p<*0.001), and compared to the 1-gram Azomite treatment (*Z=*-3.27, *p=*0.001).

### 3.3 Phyllosphere amendment increases fruit production in a dose-dependent manner (Trial 3)

To determine if the increased fruit production in PhylloStart-inoculated plants was dose-dependent, we repeated the experiment in the fall of 2020, including both the standard inoculum density (OD_600 = _0.02, High) and a lower density (OD_600 = _0.0002, Low). The trends we see in this experiment are consistent with the results from our second trial.

There was again a significant effect of PhylloStart treatment on tomato number (ANOVA; *F*(2,33)=3.417, *p=*0.045, *ges* =0.172). In this case, however, there was no observed effect of Azomite (*F*(2,33)=0.389, *p=*0.681, *ges* =0.023), nor an interaction between PhylloStart and Azomite (*F*(4,33)=0.393, *p=*0.812, *ges* =0.045). A Tukey HSD test looking specifically at the effect of PhylloStart treatment on fruit production confirmed that there was a significant increase in the total fruit produced by PhylloStart High treated plants compared to the controls (*p=*0.034, 95% C.I. = 0.44, 13.42). In the PhylloStart High treatment we saw an average of 32.2 (95% C.I. = 27.69, 36.74) tomatoes per plant, while in PhylloStart Low we see 27.1 (95% C.I. = 23.27, 30.88) and in the control plants we see 25.3 (95% C.I. = 21.67, 28.91) tomatoes per plant. As expected we did not observe any impact of PhylloStart application on tomato weight, but in contrast to our previous experiment, also did not see an effect of micronutrient supplementation on the average weight of the tomatoes produced (**Supplementary Data 1**, **Supplementary Figure 3B**).

### 3.4 Phyllosphere amendment limits subsequent colonization of a bacterial pathogen

**Figure 4:**
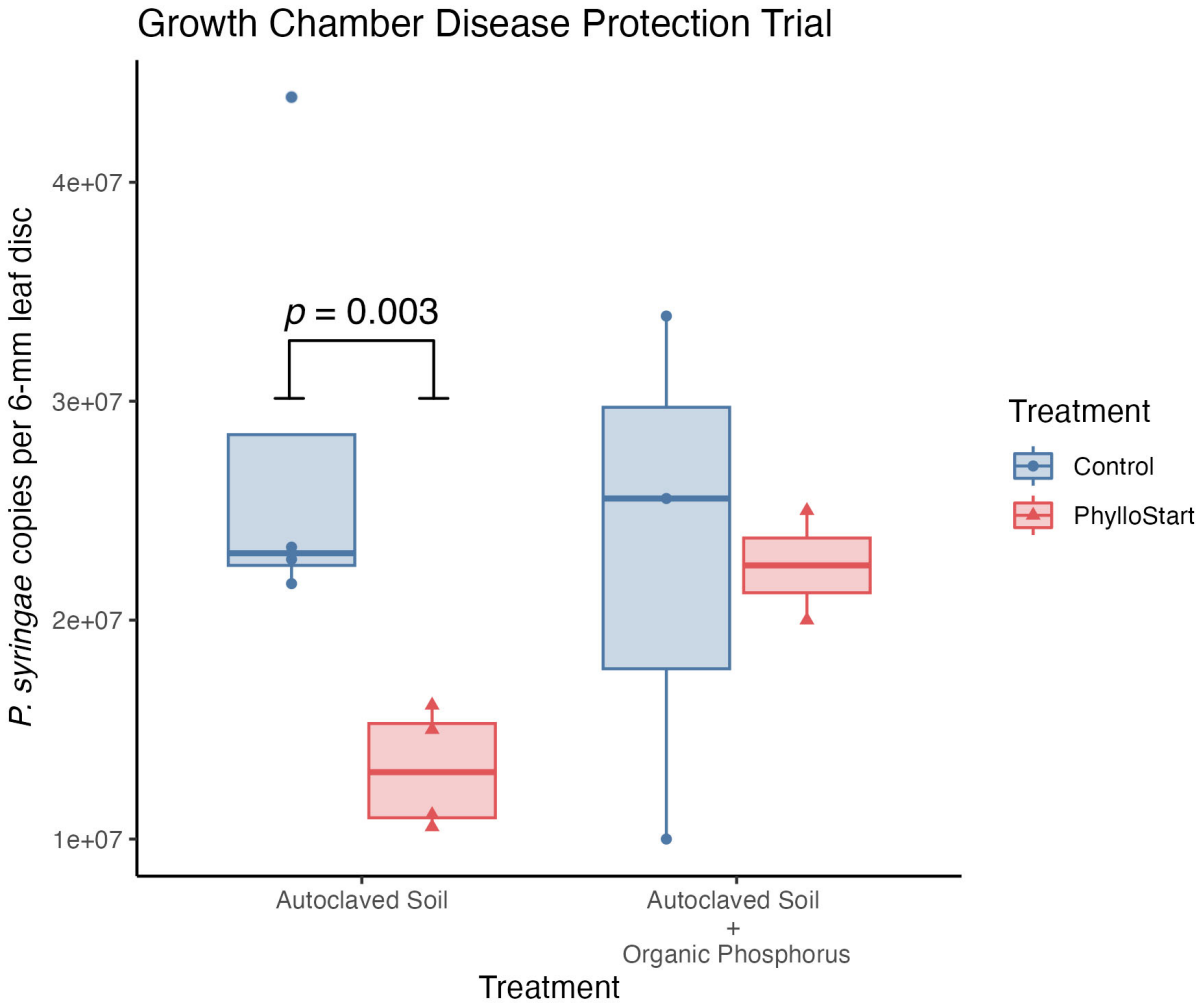
Plants inoculated with PhylloStart bacteria are protected against the establishment of the foliar pathogen *P. syringae* under low nutrient levels. However, the addition of an organic phosphorus fertilizer leads to a decrease in protection, with both the PhylloStart treated and control plants showing similar levels of pathogen development.

Previous work in tomato plants has observed that the bacteria in the phyllosphere can protect against colonization of the foliar pathogen *Pseudomonas syringae* PT23, especially under low resource conditions (Berg and Koskella, 2018). To test such an effect for PhylloStart bacteria, inoculated plants grown under either nutrient-limited conditions or high nutrient conditions were challenged with *P. syringae*. Under nutrient limitation, pathogen load was observed to be significantly lower on plants inoculated with PhylloStart than on plants inoculated with a sterile buffer control, indicating a protective effect (Wilcox Test; *t=*16.00, *p=*0.028), however, and as previously observed (Berg and Koskella, 2018), this effect disappeared among plants treated with the phosphorus fertilizer (*t=*4.00, *p=*0.8) (**Figure 4**).

### 3.5 Greenhouse plants maintain PhylloStart bacteria over time, while field plants did not under the conditions tested (Trial 3)

**Figure 5:**
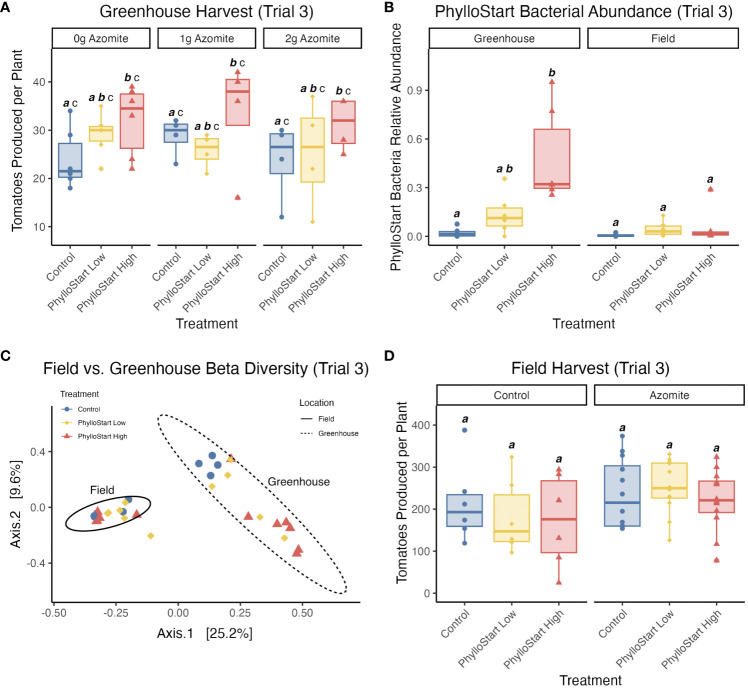
**(A)** In the third trial greenhouse-grown plants show a significant dose dependent effect of PhylloStart, where only the high inoculation density is associated with a significant increase in fruit production compared to the Controls (Control *a* vs. PhylloStart High *b*). Groups that are significantly different from each other are marked with different letters, while groups without significant differences will have the same letter. Emphasized lettering indicates comparisons across PhylloStart treatments (ie. *a*, *b*), while non-emphasized lettering indicates comparisons across Azomite treatments (ie. *c*). **(B)** Looking at the relative abundance of PhylloStart bacteria on treated and control plants, one month after inoculation, there is a significantly higher relative abundance of the PhylloStart high inoculation in the greenhouse (Control *a* vs. PhylloStart High *b*), while there are relatively few PhylloStart associated ASVs detected across any of the treatments, with no significant differences (Control, PhylloStart Low, PhylloStart High *a*).**(C)** Looking at beta diversity (Bray-Curtis Dissimilarity) across both locations, communities group both by treatment and location, with the field communities grouping together, and each greenhouse treatment separating out, with control and PhylloStart high differing, and Phyllostart low falling in between the two. **(D)** In contrast to the greenhouse data, there are no significant differences in tomatoes production between any of the PhylloStart treatments in the Field grown plants (All treatments, *a*).

To determine if the effects of PhylloStart bacteria on plant reproductive success would be seen in an environment with more potential sources of phyllosphere bacteria and/or whether early inoculation of plants changed subsequent microbiome assembly in the field, we included a field component in the third trial experiment. After inoculation, we transferred both PhylloStart-inoculated and control plants into the field. These plants were sampled concurrently with the plants from the same cohort that remained in the greenhouse (three weeks after their last inoculation), and their phyllosphere communities were sequenced. We analyzed greenhouse and field locations separately and found a significant effect of PhylloStart inoculation density on bacterial abundances in the greenhouse (Kruskal-Wallis test; *H*(2)=10.17, *p=*0.006), but no effect in the field (*H*(2)=4.07, *p=*0.13). A Dunn Post-Hoc test showed that PhylloStart High treated plants had significantly higher bacterial abundance than the control plants in the greenhouse (*Z=*3.153, *p=*0.005; **Figure 5B**).

Further, while we see that community composition is influenced by PhylloStart treatment in the greenhouse, we see no such effect in the field grown plants. When looking at a PCoA of bacterial community similarity using Bray-Curtis distance (**Figure 5C**) we see that the PhylloStart-treated greenhouse plants clearly separate out from the control plants, with the plants treated with high concentrations of PhylloStart distinct from the controls, and the plants treated with low concentrations of PhylloStart falling between the controls and the high inoculation. Meanwhile, the communities associated with the field grown plants differ from those grown in the greenhouse, but do not otherwise separate by PhylloStart treatment. When analyzing dissimilarity using an Adonis PERMANOVA, we see a significant effect of PhylloStart treatment (*F*=1.65, *p=*0.026), location; ie. field vs. greenhouse (*F*=8.47, *p=*0.001), as well as a significant PhylloStart by location interaction (*F*=1.48, *p=*0.047). When analyzing each location separately with a pairwise Adonis, we see that there are significant differences between PhylloStart high and control treated plants in the greenhouse (*F*=3.15, *p=*0.006), but no difference between PhylloStart high and low (*F*=3.15, *p=*0.084), or PhylloStart low and control (*F*=1.15, *p=*0.142) in the greenhouse. We further see no significant differences between any of the PhylloStart treatments in the field (See **Supplementary Data 1** for non-significant comparisons).

### 3.6 Early PhylloStart inoculation did not impact field-grown plants (Trial 3)

Given that the field-grown plants were colonized by many bacteria, we sought to determine whether PhylloStart treatment would still affect tomato production. Unlike in the greenhouse experiment, we did not observe any significant effect of phyllosphere amendment on yield in the field (**Figure 5D**). There was no significant effect of PhylloStart (ANOVA; *F*(2,55)=0.315, *p=*0.731, *ges* =0.045), or Azomite (*F*(3,55)=1.263, *p=*0.296, *ges* =0.045) on tomato count, and there was no significant interaction effect between the two (*F*(5,55)=0.535, *p=*0.296, *ges* =0.045). Like in the greenhouse, we saw no effect of PhylloStart on fruit weight (**Supplementary Figure 2C**). To verify these results, we performed another field trial in a subsequent year, finding broadly the same results. In this experiment, we did not see any significant difference in the total harvest yield between either the PhylloStart and Control, or PhylloStart + Azomite and Control + Azomite plants (t-test, *p=*>0.05 in all cases) (**Supplementary Data 1**, **Supplementary Figure 4**). It remains to be seen whether these amendments can provide benefits under broadacre field trials, including under biotic (powdery mildew) or abiotic (over-application of Azomite) pressures for which we observed evidence of PhylloStart benefits in the greenhouse.

## 4 Discussion

The plant microbiome is increasingly recognized for its role in shaping plant health, but most work to date has focused on below-ground associations between plant-microbe interactions. Thus far, most evidence for an impact of the above-ground, phyllosphere microbiome on their hosts has focused on disease or herbivore resistance (Mercier and Lindow, 2001; Innerebner et al., 2011; Saleem et al., 2017; Morella et al., 2019; Qin et al., 2019; Shalev et al., 2022). We sought to extend this work to determine the impact of these communities on plant growth and yield. Our initial experiment established that greenhouse-grown plants develop a significantly more abundant microbial community when inoculated with a synthetic microbial community (PhylloStart). When inoculated onto plants early in development, we found that our taxa represent the dominant members of the phyllosphere and that overall bacterial densities were far higher in amended plants relative to controls (**Figure 2B**). With two additional greenhouse studies, we determined that these microbial associations lead to a significant increase in the total number of fruit produced by greenhouse-grown tomato plants (**Figures 3**, **5A**). We also found that, in a growth chamber trial, the bacterial community provided nutrient status dependent protection from *P. syringae* establishment. In contrast, plants that were transplanted into a field environment did not appear to benefit from the initial inoculation of PhylloStart bacteria (**Figure 5D**).

Given the minimal development of the phyllosphere community in non-treated greenhouse control plants (determined by qPCR sequencing; **Figure 2B**), our findings suggest that greenhouses present an ideal location to study the effect of microbial amendments on agriculturally relevant plant traits, and support previous work finding that greenhouse-grown plants develop bacterial communities distinct from outdoor environments (Maignien et al., 2014). The extraordinarily low background levels of bacterial colonization allowed us to examine the importance of phyllosphere bacteria to plant fitness, where we found that the application of PhylloStart bacteria was associated with increased total fruit production (**Figures 3**, **5**). Further, that we do not see a fruit number/size trade-off in this study (Dombroskie et al., 2016) suggests that the microbial amendment is increasing investment in above-ground biomass, rather than simply redirecting resources from fruit size to number, as is commonly observed in seeds for example (Venable, 1992). Our results add to a body of work describing how fruit yield can be affected by both local nutrient conditions and microbial associations but extend the latter to the above ground tissues.

In contrast to the greenhouse, we did not see evidence for either establishment or impact of PhylloStart amendment in the field. In this case, PhylloStart bacteria were not found at significant abundances on these plants after a month in the field (**Figure 5B**), and their initial community structure did not seem to shape the future composition of the phyllosphere communities (**Figure 5C**). While this would seem to contradict results finding initial colonizers dominate plant microbiome assembly (Hiscox et al., 2015; Carlström et al., 2019; Debray et al., 2023), priority effects often depend on the identities of the early-colonizing species and their environments. For example, when wood disks were pre-colonized with fungi and placed in a field for six months, retention of initial colonizers in the eventual community varied between ascomycetes and basidiomycetes, and from season to season (Hiscox et al., 2015). In the tomato plant phyllosphere, PhylloStart bacteria may have been overwhelmed by dispersal from neighboring plants (Meyer et al., 2022), or from other sources. Further, the field conditions (e.g. weather, soil) during which we ran our trials may have differed from those under which we initially quantified the phyllosphere composition to design PhylloStart. Previous work has shown that community composition will depend on both plant host genotype as well as local environmental conditions (Knief et al., 2010; Ottesen et al., 2016; Lajoie and Kembel, 2021; Meyer et al., 2022). It remains possible that ‘local’ or otherwise well-adapted isolates may have yielded better performance. Further field trials under a broad spectrum of conditions and locations, as well as with larger sample sizes, are needed to determine whether bacterial amendments to the phyllosphere can potentially confer benefits to commercial field tomato production.

There are various mechanisms by which the phyllosphere community might provide the observed benefits to its host. These include: 1) altering plant hormone signaling, either directly by producing phytohormones or indirectly through the elicitation of a plant response; 2) by increasing the nutrients available to the plant either through enhanced nutrient fixation or availability; and 3) through reducing stress, either environmental or due to pathogen pressure (Karlidag et al., 2010; Adesemoye et al., 2008; Paul and Nair, 2008; Beneduzi et al., 2012; Bhattacharyya and Jha, 2012). While our study does not seek to explain the mechanism underlying observed biostimulant effects, it likely relies on a combination of these factors. However, that the effects of Azomite fertilization and PhylloStart inoculation acted primarily in an additive fashion (**Figures 3**, **5**) suggests that altered nutrient acquisition is not a particularly dominant force.

Like many phyllosphere microbiome studies, our experimental design did not specifically exclude the possible movement of bacteria to the soil (Bodenhausen et al., 2014; Bai et al., 2015; Carlström et al., 2019; Schäfer et al., 2022), and it thus remains possible that some fraction of the inoculated bacteria colonized the below-ground compartment. However, recent studies have found that phyllosphere- and rhizosphere-associated bacteria are predominately adapted for survival in their respective niches (Bai et al., 2015), and, when paired with our 16S rRNA sequencing results showing robust and long-term establishment of the community on the leaf surfaces, we think it is more likely that the effect is mediated through phyllosphere interactions directly. With this in mind, future work exploring the mechanisms of phyllosphere associated growth promotion should specifically differentiate any effects impacting above versus below ground responses to the PhylloStart bacteria, either through reciprocal translocation of the species or by physically preventing inoculation of or migration to the soil.

One specific potential mechanism for the increased reproductive success is linked to the phytohormone auxin (or IAA), which is a major regulator of plant growth, is commonly produced by bacteria inhabiting the phyllosphere (Brandl and Lindow, 1998), and has been linked to increased biomass accumulation in rice and corn (Mwajita et al., 2013; Abadi et al., 2020). In this context, increased fruit yield could be mediated by the action of auxin in decreasing flower abscission (Sexton and Roberts, 1982; Meir et al., 2010), potentially leaving more flowers available to set. Of note, we did not observe a significant change in flower number across the first trial. Using BLAST to search the genomes of the PhylloStart bacteria, we found that several members (*Bacillus wiedmannii*, *Erwinia tasmaniensis*, *Pantoea agglomerans*, and *Pantoea allii*) have matches for *idpC* (indole-3-pyruvate/phenylpyruvate decarboxylase), a key protein in auxin production (Brandl et al., 2001). Future research is needed to confirm that these bacteria can produce auxin *in planta*, and if this may explain some of their plant-beneficial effects.

It is also possible that the PhylloStart bacteria alter the plant’s response to environmental cues, allowing the plant to better optimize its growth strategy and invest more resources in reproduction. Recent work has focused on the phenomenon of microbiome-dependent ontogenic timing (MiDOT), by which the presence of certain bacterial species acts as essential cues in the developmental timing of their host organism (Metcalf et al., 2019). For example, the composition of the *Boechera stricta* (a relative of *Arabidopsis*) soil-associated bacterial community has been found to significantly alter the timing and duration of flowering (Wagner et al., 2014). Further research is needed to assess the potential role that host-associated microbes play in developmental timing.

Throughout these trials we saw no evidence of an interaction between the nutrient status of the plant and the effect of the PhylloStart bacteria; instead, the bacterial and Azomite treatments additively increased the total yield. Given these observations, we were curious if the PhylloStart community would show the nutrient dependent pathogen protection found in our lab’s previous work (Berg and Koskella, 2018). Indeed, we found, in a growth chamber experiment, that the addition of this community limited the growth of the pathogen *P. syringae* in nutrient-limited plants, but that this effect disappeared when organic phosphorus fertilizer was added (**Figure 4**). These results are in line with the stress gradient hypothesis, which posits that inter-species interactions should become more facilitative under adverse conditions (Bertness and Callaway, 1994; David et al., 2020), and highlight the important role that phyllosphere bacteria play in stress response. Indeed, previous work in this system has shown that the PhylloStart bacteria up-regulate defense responses in Arabidopsis and subsequently reducing pathogen colonization, (Mehlferber et al., 2022b).

In summary, we find that the presence of phyllosphere-associated bacteria benefit their plant host when grown in a microbially depauperate greenhouse environment, through an increase in reproductive success as measured by total fruit production, with further evidence for pathogen resistance. These results are important for understanding the role of microbial communities in host outcomes and are broadly relevant in an agricultural context where, for example, 32% of domestic and 56% of imported tomatoes in the United States are grown in greenhouses that may not provide adequate colonization of phyllosphere bacteria (Baskins et al., 2019). Further, we show that bacterial inoculation provides an additive increase in fruit production when applied with a common supplement containing micronutrients, opening avenues for further optimization of agricultural production by harnessing the biostimulant properties of phyllosphere microbes.

## Data availability statement

The data presented in the study are deposited in the following online repositories: Dryad, https://doi.org/10.5061/dryad.73n5tb333 and NCBI, accession number PRJNA852883.

## Author contributions

EM: Conceptualization, Data curation, Formal analysis, Funding acquisition, Investigation, Methodology, Supervision, Validation, Visualization, Writing – original draft, Writing – review & editing. RD: Conceptualization, Data curation, Investigation, Methodology, Writing – review & editing. AC: Conceptualization, Data curation, Investigation, Methodology, Writing – review & editing. JS: Conceptualization, Data curation, Investigation, Methodology, Writing – review & editing. GK: Data curation, Investigation, Writing – review & editing. RR: Data Curation, Investigation, Writing – review & editing. KC: Conceptualization, Writing – review & editing. JF: Conceptualization, Writing – review & editing. RK: Conceptualization, Data curation, Investigation, Methodology, Project administration, Writing – original draft, Writing – review & editing. BK: Conceptualization, Funding acquisition, Project administration, Writing – original draft, Writing – review & editing.
