# Supplementary material for: Phyllosphere microbial associations improve plant reproductive success
Source: Front Plant Sci. 2023 Dec 8;14:1273330. doi: 10.3389/fpls.2023.1273330 (PMC10739325; doi:10.3389/fpls.2023.1273330)
Supplement: Supplementary file 3 [file DataSheet_1.zip › Supplementary Figures.pdf]

Supplementary Material

Phyllosphere microbial associations improve plant reproductive success

Elijah C. Mehlferber[1*], Reena Debray[1], Asa E. Conover[1], Julia K. Sherman[1], Griffin Kaulbach[3], Robert Reed[5], Kent F. McCue[2], Jon E. Ferrel[4,5], Rajnish Khanna[5,6,7], and Britt Koskella[1,8]

*** Correspondence:**
Elijah Mehlferber
emehlferber@berkeley.edu

Britt Koskella
bkoskella@berkeley.edu

1 Supplementary Figures

	Trial 1	Trial 2	Trial 3	
**Location**	Greenhouse	Greenhouse	Greenhouse	Field
**Bacterial Treatments**	PhylloStart at OD600=0.02	PhylloStart at OD600=0.02	PhylloStart at OD600=0.02 and OD600=0.0002	PhylloStart at OD600=0.02 and OD600=0.0002
**Azomite Treatments**	5% Granular<br>1g Ultrafine<br>5% Granular + 1g Ultrafine	5% Granular + 1g Ultrafine<br>5% Granular + 2g Ultrafine<br>5% Granular + 3g Ultrafine	5% Granular + 1g Ultrafine<br>5% Granular + 2g Ultrafine	5% Granular + 1g Ultrafine<br>5% Granular + 3g Ultrafine
**Replicates per Treatment**	1. Control (MgCl Spray + No Azomite) *(n=10)*<br>2. PhylloStart Only *(n=10)*<br>3. 5% Granular *(n=10)*<br>4. 1g Ultrafine *(n=10)*<br>5. 5% Granular + 1g Ultrafine *(n=10)*<br>6. 5% Granular + 1g Ultrafine + PhylloStart *(n=10)*	1. Control (MgCl Spray + No Azomite)*(n=3)*<br>2. 5% Granular + 1g Ultrafine *(n=3)*<br>3. 5% Granular + 2g Ultrafine *(n=3)*<br>4. 5% Granular + 3g Ultrafine *(n=3)*<br>5. PhylloStart Only *(n=3)*<br>6. 5% Granular + 1g Ultrafine + PhylloStart *(n=3)*<br>7. 5% Granular + 2g Ultrafine + PhylloStart *(n=3)*<br>8. 5% Granular + 3g Ultrafine + PhylloStart *(n=3)*	1. Control (MgCl Spray + No Azomite) *(n=6)*<br>2. 5% Granular + 1g Ultrafine *(n=4)*<br>3. 5% Granular + 2g Ultrafine *(n=4)*<br>4. PhylloStart High Density *(n=6)*<br>5. PhylloStart Low Density *(n=6)*<br>6. 5% Granular + 1g Ultrafine + PhylloStart High *(n=4)*<br>7. 5% Granular + 2g Ultrafine + PhylloStart Low *(n=4)*<br>8. 5% Granular + 1g Ultrafine + PhylloStart High *(n=4)*<br>9. 5% Granular + 2g Ultrafine + PhylloStart Low *(n=4)*	1. Control (MgCl Spray + No Azomite) *(n=6)*<br>2. 5% Granular + 1g Ultrafine *(n=4)*<br>3. 5% Granular + 2g Ultrafine *(n=4)*<br>4. PhylloStart High Density *(n=6)*<br>5. PhylloStart Low Density *(n=6)*<br>6. 5% Granular + 1g Ultrafine + PhylloStart High *(n=4)*<br>7. 5% Granular + 3g Ultrafine + PhylloStart Low *(n=4)*<br>8. 5% Granular + 1g Ultrafine + PhylloStart High *(n=4)*<br>9. 5% Granular + 3g Ultrafine + PhylloStart Low *(n=4)*
**Main Findings**	1. PhylloStart efficiently colonizes the leaves<br>2. The leaves of non-PhylloStart treated greenhouse plants have low bacterial abundance	1. PhylloStart treated plant **produce significantly more fruit** than non-inoculated controls, with no change in fruit weight<br>2. Azomite treatment also **significantly increases fruit production, as well as fruit weight**	1. Different densities of PhylloStart application lead to different amounts of leaf colonization<br>2. PhylloStart High Density **significantly increases fruit production**, PhylloStart Low Density does not	1. PhylloStart does not persist after plants are transplanted to the field<br>2. No evidence for priority effects in field plants, community composition over time is unchanged<br>3. No evidence that PhylloStart inoculation leads to increased fruit production in the field, maybe due to sufficient exposure to natural phyllosphere bacteria

Sup Fig 1. Outline of the different treatments and replicates used across experiments, as well as the main findings from each experiment.

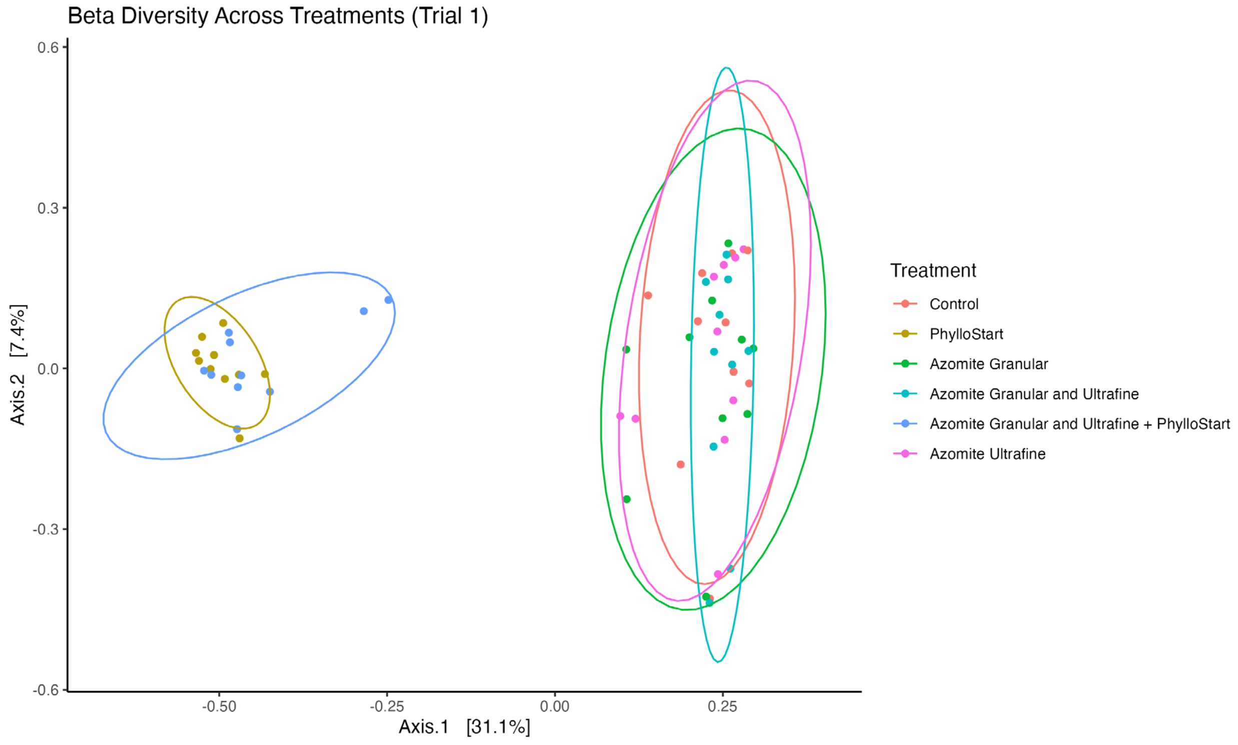

Sup Fig 2. Beta diversity measured as Bray-Curtis Distance between PhylloStart treated and control plants, with the Azomite treatments included. We see no effect of Azomite treatment on Bray-Curtis Distance (Sup Dat 1).

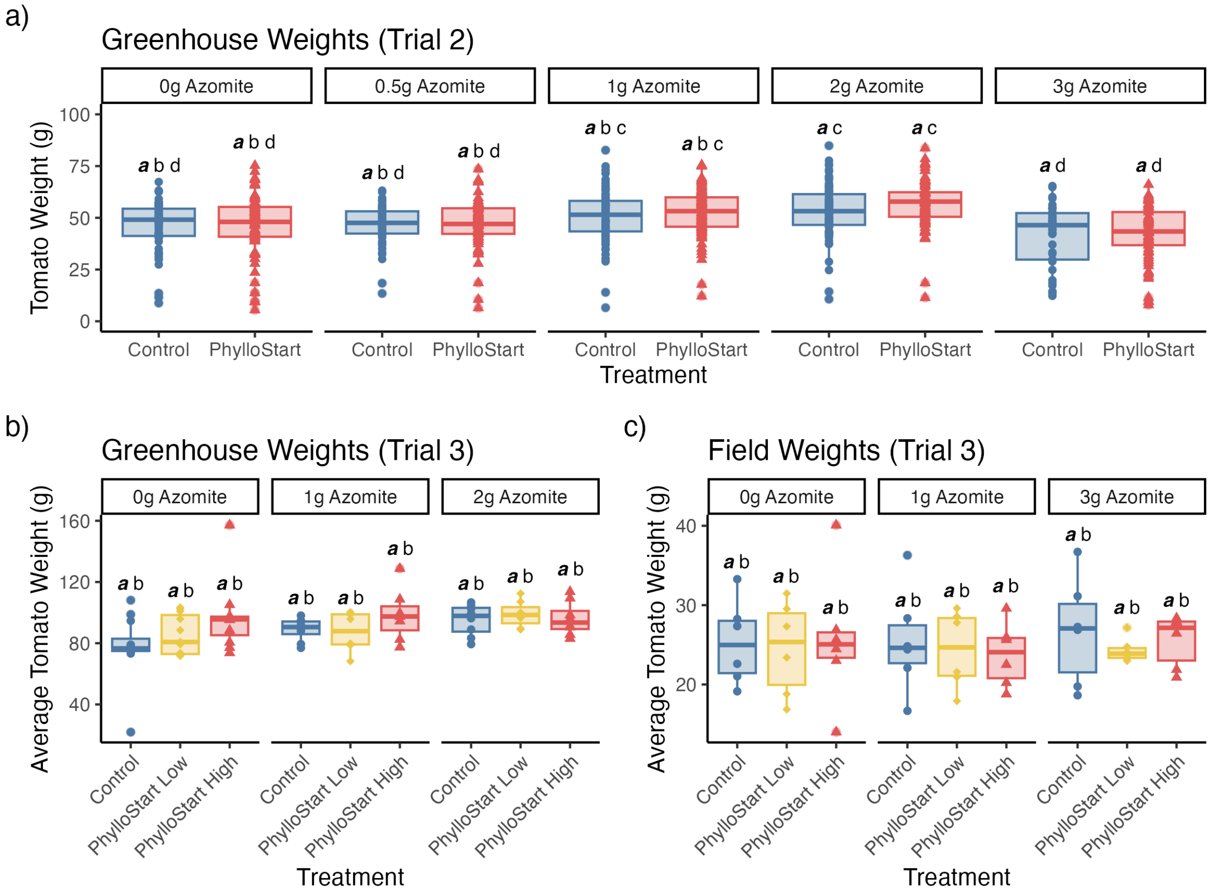

Sup Fig 3. Tomato weights as measured throughout the greenhouse and field trials. Groups that are significantly different from each other are marked with different letters, while groups without significant differences will have the same letter. Emphasized lettering indicates comparisons across PhylloStart treatments, while non-emphasized lettering indicates comparisons across Azomite treatments. **(A)** In the second greenhouse trial, several individual tomatoes from each plant were collected and weighed. We see no significant effect of PhylloStart treatment on tomato weight, though there are significant differences among several Azomite treatments (Sup Dat 1). **(B)** In the third greenhouse trial, average tomato weight was determined based on the number of total fruit and the total harvest weight per plant. Again, there was no significant effect of PhylloStart on tomato weight, and in contrast to the previous trial there was no significant effect of Azomite treatment (Sup Dat 1). **(C)** Likewise, there was no significant effect of PhylloStart or Azomite on tomato weight in the field trial (Sup Dat 1).

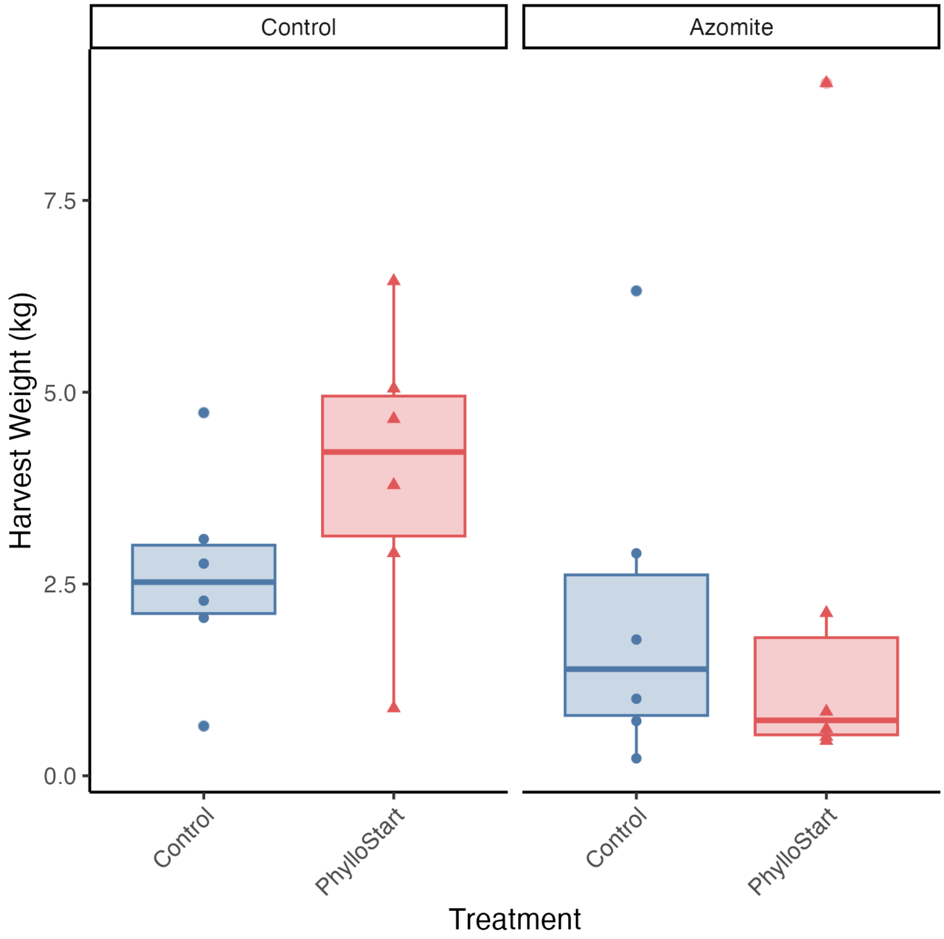

Sup Fig 4. In order to confirm that there was no effect of PhylloStart treatment on the number of tomatoes in the field, the experiment was repeated in a subsequent year. Again, there was no significant impact of PhylloStart treatment, or in this case micronutrient supplementation on the total harvest weight at the end of the field season.
