# Supplementary material for: Phyllosphere microbial associations improve plant reproductive success
Source: Front Plant Sci. 2023 Dec 8;14:1273330. doi: 10.3389/fpls.2023.1273330 (PMC10739325; doi:10.3389/fpls.2023.1273330)
Supplement: Supplementary file 3 [file Supplementary Data.PDF › Supplementary Data.PDF]

Supplemental_Data_1

Elijah Mehlferber

2023-05-14

Load Packages

```
library(tidyverse)
library(phyloseq)
library(dada2)
library(reltools)
library(patchwork)
library(data.table)
library(readxl)
library(reshape2)
library(nlme)
library(vegan)
library(ggplot2)
library(ggpattern)
library(viridis)
library(rmarkdown)
library(ggpubr)
library(rstatix)
library(grid)
library(gridExtra)
library(pairwiseAdonis)
library(mirlyn)
library(speedyseq)
library(robustlmm)
library(car)
library(MESS)
library(ggview)
library(conflicted)

conflicts_prefer(phyloseq::transform_sample_counts)
conflicts_prefer(phyloseq::psmelt)
conflicts_prefer(dplyr::recode)
conflicts_prefer(reshape2::melt)
conflicts_prefer(dplyr::filter)
conflicts_prefer(phyloseq::filter_taxa)

g_legend<-function(a.gplot){
  tmp <- ggplot_gtable(ggplot_build(a.gplot))
  leg <- which(sapply(tmp$grobs, function(x) x$name) == "guide-box")
  legend <- tmp$grobs[[leg]]
  return(legend)}

summarySE2 <- function(x, num_var){
  num_var <- enquo(num_var)

  x %>%
    summarize(avg = mean(!!num_var), n = n(),
              sd = sd(!!num_var), se = sd/sqrt(n))
}

options(scipen=999)

path <- "/Users/Lij_Mehlferber/Documents/Research/Grad_Research/Phyllostart Paper/01_Follow-Up/Frontiers_Submission/Data_For_Dryad/"
```

Main Figures

Figure 2

```
# Load Data
ps.Greenhouse_Trial_Fall_2019 <- readRDS(paste0(path, "Trial_1_Greenhouse_PhyloseqObject_relAbund.rds"))

df.Greenhouse_Trial_Fall_2019.abs <- readRDS(paste0(path, "Trial_1_Greenhouse_Dataframe_absAbund.rds"))

pal2 <- c("#4e79a7", "#e15759")

# Relative Abundance

NewSynComNames <- c("Pantoea agglomerans/allii",
                    "Erwinia tasmaniensis",
                    "Pseudomonas asturiensis/rhizosphaerae",
                    "Pseudomonas rhodesiae",
                    "Pseudomonas moraviensis",
                    "Massilia aurea",
                    "Brevibacterium frigoritolerans",
                    "Microbacterium oleivorans",
                    "Curtobacterium herbarum/pusillum",
                    "Pseudomonas asturiensis/rhizosphaerae",
                    "Frigoribacterium endophyticum",
                    "Curtobacterium herbarum/pusillum",
                    "Rathayibacter festucae",
                    "Bacillus wiedmannii",
                    "Exiguobacterium sibiricum"
                    )

BacteriaOfInterest <- c(unique(NewSynComNames), "Proteobacteria", "Actinobacteriota", "Firmicutes", "Bacteroidota", "Cyanobacteria", "Other")
# Some bacteria are in there twice due to how I had to name them, so I'm using unique to remove them here because these will become the factor levels

library(RColorBrewer)

palette = c(rev(brewer.pal(9, "Oranges"))[3:8], rev(brewer.pal(9, "Blues"))[3:7], rev(brewer.pal(9, "Greens"))[4:5], brewer.pal(9, "Reds")[2], brewer.pal(9, "Blues")[2], brewer.pal(9, "Greens")[2], brewer.pal(9, "Greys")[4], brewer.pal(9, "Greys")[3],   "#ffffff")

names(palette) = BacteriaOfInterest


ps.Greenhouse_Trial_Fall_2019.rel <- transform_sample_counts(ps.Greenhouse_Trial_Fall_2019, function(OTU) OTU/sum(OTU))

df.Greenhouse_Trial_Fall_2019.rel <- psmelt(ps.Greenhouse_Trial_Fall_2019.rel) %>%
  mutate(Species = case_when(Species =="Pantoea aurea" ~ "Massilia aurea", .default = Species)) %>%
  mutate(Species= ifelse(is.na(Species),Phylum,Species)) %>%
  mutate(Species= ifelse(Species %in% BacteriaOfInterest, Species, "Other")) %>%
  mutate(SynCom = ifelse(Species %in% NewSynComNames, "SynCom", "Non SynCom")) %>%
  group_by(Sample_ID, Species, Treatment, SynCom) %>%
  summarise(TotAbundance = sum(Abundance), .groups = "drop") %>%
  filter(Treatment %in% c("Control", "Control+Bacteria")) %>%
  mutate(Treatment=dplyr::recode(Treatment, 'Control'='Control', 'Control+Bacteria'='PhylloStart')) %>%
  mutate(Species = factor(Species, levels = BacteriaOfInterest))

p.RelAbund <- ggplot(df.Greenhouse_Trial_Fall_2019.rel, aes(x=Sample_ID, y=TotAbundance, fill=Species, color=Species, pattern=SynCom)) +
  geom_bar(stat = "identity", color="black") +
  facet_grid(~ Treatment, scales = "free", space = "free", drop = TRUE) +
  theme_classic() +
  scale_fill_manual(values=palette) +
  ylab("Relative Abundance") +
  theme(axis.title.x=element_blank(),
        axis.text.x=element_blank(),
        axis.ticks.x=element_blank()) +
  ggtitle("Relative Abundance of Greenhouse ASVs (Trial 1)") +
  theme(plot.title = element_text(hjust = 0.5)) +
  theme(text = element_text(size = 15))

p.RelAbund %>% ggview(width = 13, height = 5)

# Absolute Abundance

p.AbsAbund <- df.Greenhouse_Trial_Fall_2019.abs %>%
  group_by(Sample_ID, Treatment) %>%
  summarize(sum(TotalAbundance)) %>%
  ggplot(aes(x=Sample_ID, y=`sum(TotalAbundance)`, fill = Treatment)) +
  geom_bar(stat = "identity", color="black") +
  facet_grid(~ Treatment, scales = "free", space = "free", drop = TRUE) +
  theme_classic() +
  scale_fill_manual(values=pal2) +
  ylab("Absolute Abundance (Copies/g Leaf Mass)") +
  theme(axis.title.x=element_blank(),
        axis.text.x=element_blank(),
        axis.ticks.x=element_blank()) +
  ggtitle("Greenhouse Bacterial 16s rRNA Copies (Trial 1)") +
  theme(plot.title = element_text(hjust = 0.5)) +
  theme(text = element_text(size = 15))
```

```
## `summarise()` has grouped output by 'Sample_ID'. You can override using the
## `.groups` argument.
```

```
# Legends
Legend_1 <-
  df.Greenhouse_Trial_Fall_2019.rel %>%
  filter(SynCom %in% "SynCom") %>%
  ggplot(aes(x=Sample_ID, y=TotAbundance, fill=Species)) +
  geom_bar(stat="identity", color="black") +
  guides(fill=guide_legend(ncol=1)) +
  scale_fill_manual(values = palette[1:14], name = "PhylloStart") +
  theme(text = element_text(size = 15)) +
  theme(text = element_text(face="italic"))

Legend_2 <-
  df.Greenhouse_Trial_Fall_2019.rel %>%
  filter(SynCom %in% "Non SynCom") %>%
  ggplot(aes(x=Sample_ID, y=TotAbundance, fill=Species)) +
  geom_bar(stat="identity", color="black") +
  guides(fill=guide_legend(ncol=1)) +
  scale_fill_manual(values = palette[14:19] , name = "Non-PhylloStart") +
  theme(text = element_text(size = 15))

layout.fig2 <- "
AAAAABBBBB
AAAAABBBBB
###C##D###
"
layout.fig2 <- "
AAAAA#####
AAAAACC###
AAAAACC###
AAAAA#####
BBBBBD####
BBBBB#####
BBBBB#####
BBBBB#####
"

Fig_2 <- ((p.RelAbund + theme(legend.position = "None")) + labs(tag = "a)")) +
  ((p.AbsAbund + theme(legend.position = "None")) + labs(tag = "b)")) +
  g_legend(Legend_1) + g_legend(Legend_2) + plot_layout(design = layout.fig2)

Fig_2 %>% ggview(width = 13, height = 10)
```

Figure 3

```
InputFile <- "Trial_2_Greenhouse_Fruit_Count.csv"

pal2 <- c("#4e79a7", "#e15759")

Harvest.Spring.2020 <- paste0(path, InputFile) %>%
  read_csv(col_names = TRUE) %>%
  arrange(Azomite) %>%
  mutate(Bacteria = case_when(
    Bacteria %in% "Bacteria" ~ "PhylloStart",
    Bacteria %in% "Control" ~ "Control"
  ))
```

```
## New names:
## Rows: 30 Columns: 6
## ── Column specification ──────────────────────────────── Delimiter: "," chr
## (3): ID, Treatment, Bacteria dbl (3): ...1, Azomite, Total_Fruit
## ℹ Use `spec()` to retrieve the full column specification for this data. ℹ
## Specify the column types or set `show_col_types = FALSE` to quiet this message.
## • `` -> `...1`
```

```
Harvest.Spring.2020$Azomite <- rep(c("0g Azomite", "0.5g Azomite", "1g Azomite", "2g Azomite", "3g Azomite"), each = 6)
Harvest.Spring.2020$Azomite <- factor(Harvest.Spring.2020$Azomite, levels = c("0g Azomite", "0.5g Azomite", "1g Azomite", "2g Azomite", "3g Azomite"))

Harvest.Spring.2020$Bacteria <- factor(Harvest.Spring.2020$Bacteria, levels = c("Control", "PhylloStart"))

Fig3 <- ggplot(Harvest.Spring.2020, aes(x=Bacteria, y=Total_Fruit, fill=Bacteria)) +
  geom_boxplot(aes(color = Bacteria), alpha=0.3) +
  geom_point(aes(color = Bacteria, shape = Bacteria)) +
  facet_grid(~ Azomite, scales = "free") +
  scale_fill_manual(name = "Treatment", labels = c("Control", "PhylloStart"), values = pal2) +
  scale_color_manual(name = "Treatment", labels = c("Control", "PhylloStart"), values = pal2) +
  scale_shape_manual(name = "Treatment", values=c(16, 17), labels= c("Control", "PhylloStart")) +
  theme_classic() +
  ylab("Tomatoes Produced per Plant") +
  theme(axis.text.x = element_text(angle = 45, hjust = 1)) +
  ylim(10,40) +
  xlab("Treatment") +
  ggtitle("Greenhouse Harvest (Trial 2)")

Fig3 %>% ggview(width = 5, height= 5)
```

Figure 4

[illegible]
